# Genomics and Epigenomics Approaches for the Quantification of Circulating Tumor DNA in Liquid Biopsy: Relevance of a Multimodal Strategy

**DOI:** 10.3390/ijms262210982

**Published:** 2025-11-13

**Authors:** Elisa De Paolis, Alessia Perrucci, Gabriele Albertini Petroni, Alessandra Conca, Matteo Corsi, Andrea Urbani, Angelo Minucci

**Affiliations:** 1 Departmental Unit of Molecular and Genomic Diagnostics, Genomics Core Facility, Gemelli Science and Technology Park (G-STeP), Fondazione Policlinico Universitario A. Gemelli IRCCS, 00168 Rome, Italy; alessia.perrucci@guest.policlinicogemelli.it (A.P.); gabriele.albertinipetroni01@icatt.it (G.A.P.); alessandra.conca@guest.policlinicogemelli.it (A.C.); matteo.corsi@guest.policlinicogemelli.it (M.C.); angelo.minucci@policlinicogemelli.it (A.M.); 2 Clinical Chemistry, Biochemistry and Molecular Biology Operations (UOC), Fondazione Policlinico Universitario A. Gemelli IRCCS, 00168 Rome, Italy; andrea.urbani@policlinicogemelli.it; 3 Department of Basic Biotechnological Sciences, Intensivological and Perioperative Clinics, Catholic University of Sacred Heart, 00168 Rome, Italy

**Keywords:** genomics, epigenomics, transcriptomics, omics, ctDNA, cTF, cfDNA, liquid biopsy, methylation, NGS

## Abstract

The adoption of liquid biopsy approaches in clinical practice has triggered a significant paradigm shift in the diagnostic, prognostic, and predictive outcomes for cancer patients. Circulating tumor DNA (ctDNA) is considered a valuable biomarker for monitoring tumor burden and its mutational dynamics. In this context, not all cell-free DNA (cfDNA) molecules are derived from tumor cells. Furthermore, due to tumor heterogeneity, not all ctDNA molecules contain cancer-associated alleles, complicating the direct quantification of the circulating tumor allele fraction (cTF) within the total cfDNA. Cancer arises from the accumulation of multiple genetic and epigenetic changes. Each of these molecular features can be exploited as the basis of methodological strategies used in ctDNA quantification. Different layers of omics data, from genomics, evaluating mutational analysis of somatic single-nucleotide variants and copy number alterations, to epigenomics, primarily consisting of the evaluation of methylation profiles and fragmentation patterns, can be used for this purpose. Some of these approaches can be effective in a multi-modal manner. To date, the quantification approaches for estimating cTF vary enormously, making direct comparisons and an assessment of their translational value challenging. Moreover, the lack of regulatory approval for many of these assays is a critical barrier to their widespread clinical adoption. This review explores the different omics approaches described for ctDNA quantification, outlining strengths and limitations, and highlighting their valuable applications in clinical settings.

## 1. Introduction

Liquid biopsy (LBx) involves the comprehensive evaluation of circulating biomolecules as circulating tumor cells (CTCs), cell-free DNA (cfDNA), cell-free RNA (cfRNA), proteins, lipids, and metabolites [1]. LBx has gained an increasingly important role in various clinical contexts, notably in non-invasive prenatal testing (NIPT) and as a significant non-invasive biomarker in oncology [2]. Focusing on cfDNA, an increase in its total content in body fluids can be linked to several physiological or para-physiological conditions, including pregnancy and transplantation, as well as pathological states like diabetes, inflammation, sepsis, and autoimmune processes [3,4,5]. Circulating tumor DNA (ctDNA) constitutes the fraction of the total cfDNA that is derived from tumor cells [6]. The current and potential clinical applications of LBx in oncology range from the early detection and risk stratification of cancer to monitoring treatment response, which allows for the detection of molecular resistances and minimal residual disease (MRD) [2]. Additionally, an ambitious approach involves multicancer early detection (MCED), where LBx serves as a marker for the early identification of a malignant event in seemingly healthy individuals [7]. One of the key advantages of ctDNA analysis is its ability to provide real-time monitoring and to reflect the dynamic genomic landscape of cancer, offering insights into tumoral heterogeneity without the need for solid biopsies of invasive tissue (TBx) [8,9]. Furthermore, total ctDNA levels have been proven to have prognostic value and are strongly correlated to clinical features and outcomes [10,11,12,13,14].

In the last decade, the establishment of the NIPT, with its evaluation of fetal cfDNA in maternal plasma, has supported and promoted the parallel improvement of LBx, particularly from a methodological standpoint [15]. Despite long-standing efforts, the widespread clinical application of LBx has been slow in most contexts. This is primarily due to a few major challenges, such as the complexity of adopting and standardizing established methods and the need for a critical interpretation of the molecular results. In the plethora of clinical applications and studies, the effective discrimination of the ctDNA ratio from the cfDNA background is critical for obtaining reliable and reproducible results, especially when interpreting a true negative genotyping. In LBx, the absence of tumor-specific alterations may genuinely reflect the tumor genotype (i.e., true negative) or may be a result of insufficient ctDNA shedding, low content, or inadequate detection sensitivity (i.e., false negative) [6]. For this reason, the Food and Drug Administration (FDA) label for approved LBx applications recommended a reflex test for TBx for negative LBx results across several tumor types [16].

With these premises, the accurate estimation of the ctDNA rate, and thus the circulating tumor allele fraction (cTF), is a challenging topic when it comes to ensuring the actionability of molecular findings from LBx. To advance this field, an improved understanding of the biological properties of cfDNA has accelerated the development of new diagnostic and methodological tools.

To date, the literature lacks an established and well-accepted consensus on the best methodological practice for ctDNA quantification. This review describes the main approaches and methodologies currently available to estimate the ctDNA fraction, while also providing an overview of the biological characteristics of ctDNA that are relevant to the purpose of this manuscript. The ultimate goal is to provide clinicians and experts with a valuable reference of the current state of the art, underscoring the need for clear guidelines on this topic.

## 2. Biological Characteristics of cfDNA and ctDNA

cfDNA refers to fragmented DNA molecules that circulate in body fluids like blood plasma and serum. These fragments originate from various physiological processes, including apoptosis, necrosis, and active secretion [17,18,19,20]. Subcomponents of cfDNA primarily consist of nuclear and mitochondrial DNA. Minor biological fractions are also present, such as circular DNA (eccDNA), microDNA, and DNA derived from viruses, bacteria, and food (plants and meat) [21,22,23]. ctDNA is a fraction of cfDNA specifically derived from tumor cells. It ideally carries the same genetic background as the primary tumor and metastatic lesions, making it a non-invasive source of cancer molecular information [2]. Due to the heterozygosity and the tumor heterogeneity, not all ctDNA molecules at a specific locus contain the mutated tumor allele. For this reason, we refer to the term cTF as the fraction of cfDNA that originates from the tumor and contains a tumor-specific allele [7]. Therefore, cTF comprises ctDNA fragments containing tumor-specific somatic single-nucleotide variants (SNVs), copy number alterations (CNAs), or an abnormal epigenomics profile. The cTF is commonly used as a measure of ctDNA signal in many LBx cancer detection tests. Typically, cfDNA fragments range in size from 100 to 800 base pairs, with a prominent peak around 160–180 base pairs, which corresponds to the length of DNA wrapped around a nucleosome [24]. Some individual cfDNA molecules, however, can be much larger, exceeding 20–30 kb [25,26,27]. The ctDNA fragments are often shorter, typically around 130–150 base pairs [24]. The concentration of cfDNA in the blood of healthy subjects ranges between 0 and 100 ng/mL [3,25,26,27]. Studies on tissue-specific methylation patterns in healthy individuals have shown that most cfDNA is of hematopoietic origin, with a fraction also originating from solid tissues as vascular endothelial cells, neurons, and hepatocytes [28,29,30]. In cancer patients, cfDNA levels can rise more than 20-fold, sometimes exceeding 1000 ng/mL, with the ctDNA fraction ranging from 0.1 to 89%. This proportion may increase with disease progression [25,31,32,33,34]. The variability in ctDNA levels depends on several factors, including the tumor type and stage. High ctDNA shedding is observed in some advanced cancers (e.g., non-small cell lung cancer, colorectal cancer, breast cancer, etc.), while lower release occurs in others (e.g., primary brain, renal, and thyroid cancers) [31,35]. Early-stage tumors or benign lesions typically shed lower amounts of cfDNA, which poses a challenge for the clinical use of liquid biopsy in these settings [36,37]. An additional consideration for cfDNA/ctDNA detection is the balance between their release and clearance, which is primarily mediated by the liver and kidneys. In healthy individuals, efficient clearance mechanisms partially account for the low cfDNA levels. The increased content of necrotic/apoptotic cells and the chronic inflammatory context affect these clearance mechanisms, which is a contributing factor to the increased cfDNA/ctDNA concentrations [23].

These biological features and the differences between cfDNA and ctDNA are the conceptual basis for developing and adopting different strategies to estimate the tumor fraction rate of cfDNA.

## 3. Circulating Tumor Fraction Estimation Technologies

First and foremost, it is important to emphasize that the ability to accurately estimate the cTF from total cfDNA begins with the proper collection of bodily fluid and the efficient isolation of nucleic acids. These pre-analytical steps are crucial for cTF estimation because they can significantly affect the generation of background noise, thereby limiting the probability of analyzing a true tumor-derived signal [1]. A critical analysis of these pre-analytical variables and their impact on LBx performance is beyond the scope of this review, and valuable literature is available [38,39,40].

To the best of our knowledge, there is currently no well-recognized method to discriminate between ctDNA and cfDNA during the nucleic acid isolation step, primarily because both types of nucleic acids share similar characteristics and are extracted under the same conditions. A number of molecular and genomic characteristics can be derived from cfDNA and used to infer the cTF, each with its own performance metrics and limitations. The most common cfDNA post-isolation approaches exploit the evaluation of the following: (1) tumor-specific mutations, (2) quantitative changes, (3) methylation patterns, and (4) fragment profiles.

Most of the technologies described here are based on next-generation sequencing (NGS) coupled with specialized bioinformatics pipelines for data analysis. The success of the NGS approach in estimating cTF relies on its sensitivity and specificity to detect the often low levels of ctDNA. It also allows for the identification of a wide range of rare genomic alterations, including SNVs and CNAs, across the entire genome or within targeted regions. Just as in somatic variants’ identification from a solid biopsy, unique molecular identifiers (UMIs) are incorporated into the common NGS library preparation workflows to reduce background noise and enable the detection of rare variants, especially with low amounts of starting ctDNA. In addition to genetic features, epigenetic modifications have also gained attention as a valuable source of cTF information. Generally speaking, epigenetic signatures have emerged as a superior marker, especially in early-stage settings, when compared with genetic screening [41].

Significant differences in the adoption and optimization of specific approaches for cTF estimation depend on the type of underlying tumor-derived molecular alteration being analyzed (i.e., SNVs, CNAs, or epigenetic features).

### 3.1. Genomics Analysis of the Circulating Tumor Fraction: Somatic Single-Nucleotide Variants

Early attempts to infer the tumor fraction of cfDNA relied on a personalized approach, where a specific somatic single-nucleotide variant (SSNV) identified in a patient’s TBx was subsequently monitored in their plasma samples over time [42]. Using this targeted approach, the cTF was typically inferred according to the variant allele frequency (VAF) or mutant allele frequency (MAF). These parameters represent the percentage of NGS reads that contain the mutant allele relative to the total number of reads at a given locus [43]. The AF-based approach is a robust and useful method for clinical purposes, allowing for the dynamic monitoring of one or a few given SSNVs. The clinical value of AF as an independent prognostic and predictive biomarker has been well-documented in numerous studies [9,44,45,46,47,48].

Methods for inferring cTF using SSNVs include digital PCR (i.e., droplet digital PCR (ddPCR) and beads, emulsions, amplification, and magnetics (BEAMing)), optimized quantitative real-time PCR (AS-NEPB-PCR, PNA-LNA PCR clamp, COLD-PCR), and targeted deep sequencing (>5000× coverage) with or without error suppression [31,49,50,51]. These approaches are sensitive and fast, capable of quantifying mutant DNA copies down to a 0.01% AF. As a proof-of-concept study, the TRACERx clinical trial used a targeted multiplex PCR approach to monitor ctDNA after whole-exome sequencing genotyping of TBx. The study demonstrated that in 92.9% of early-stage lung cancer relapse cases, ctDNA was detected 70 days before clinical computed tomography (CT) scan confirmation [17].

However, this type of targeted approach requires both TBx and LBx analyses and, in some cases, germline sequencing data. This makes it time-consuming, relatively expensive due to the need for a patient-specific custom design, and not always feasible. In addition, it does not overcome issues related to the unviability of TBx in some clinical contexts (e.g., small biopsy, challenging tissue site location, etc.) and limits the monitoring of tumor evolution and heterogeneity over time [8]. It is also possible to adopt broader, tissue-agnostic NGS multi-gene panels. These approaches use optimized analytical and bioinformatics workflows that overcome critical biases, such as background noise from sources like clonal hematopoiesis of indeterminate potential (CHIP) [52]. In these TBx-free NGS approaches, the evaluation of cTF at multiple loci is combined into a single estimation [7]. To increase assay sensitivity, several optimized strategies have been developed to enrich the proportion of tumor-derived DNA before targeted deep NGS. In 2012, Forshew et al. described the tagged-amplicon deep sequencing technology (TAm-Seq) as a highly sensitive approach able to detect low-frequency mutations (as low as 2%) in cfDNA from patients with high-grade serous ovarian cancer [53]. Since then, several high-sensitivity NGS-based methods have been developed, such as the enhanced TAm-Seq (eTAm-Seq™) technology [54] and the error-suppressed multiplexed deep sequencing method. This method distinguishes true mutations in ctDNA from amplification artifacts and sequencing errors, increasing detection sensitivity to as low as 0.02% [55]. Cancer personalized profiling by deep sequencing (CAPP-Seq) is another approach designed for improved sensitivity and specificity (up to 0.02%) [56]. In the single-molecule amplification and resequencing technology (cSMART) assay, each cfDNA molecule is uniquely barcoded and universally amplified. The amplification products are then circularized and reamplified in a target-specific manner to enrich a pool of target sequences containing hotspot mutations [57,58]. The cSMART analysis has been successfully adopted in the DYNAMIC study, which proved that the clearance of ctDNA in patients with lung cancer accurately reflects real-time tumor burden in response to therapy. In this study, the ctDNA rate was calculated by multiplying the MAF% by the cfDNA concentration [59].

For estimating ctDNA rates using large, high-sensitivity NGS panels, the maximum somatic allele frequency (MSAF) is often used as a surrogate for ctDNA calculation. Most cfDNA molecules are wild-type; therefore, the median MAF% of somatic alterations is generally low (<0.5%). To infer the ctDNA fraction, parameters such as the higher sum VAF of genomic alterations in ctDNA (%ctDNAsum) and the higher maximum VAF of genomic alterations in ctDNA (%ctDNAmax) have been proposed. The %ctDNAsum represents the sum of the VAF% of the individual alterations identified in the ctDNA (excluding the variants of unknown significance). The %ctDNAmax represents the maximum VAF% of the alterations identified in the ctDNA (excluding the variants of unknown significance). Several studies correlated high loads of genomic alterations, represented by high %ctDNAsum and %ctDNAmax values, with clinical outcomes in various oncological contexts [60,61,62,63,64,65,66]. The use of such a parameter for the ctDNA% estimation is part of several commercial kits, such as the ctTSO500HT (Illumina, San Diego, CA, USA). While the use of large, targeted gene panels expands the ability to evaluate tumor dynamics over time with great sensitivity, the limitations related to the choice of the gene panel content still remain [67].

In addition to a targeted deep sequencing approach, an example of optimized low-coverage genome-wide NGS approaches has been described. One such example is the GEMINI (GEnome-wide Mutational Incidence for Non-Invasive detection of cancer) tool, which consists of sequencing individual cfDNA molecules to enrich for ctDNA somatic mutations without requiring a matched blood and tumor sequencing [68]. Broader sequencing approaches for cTF quantification using SSNV include whole-exome sequencing (WES) and whole-genome sequencing (WGS) (with a sensitivity of 5–10%). These methods ideally allow for the investigation of unknown and novel acquired somatic variants, maximizing the study of tumor evolution. However, the need for high-coverage sequencing to detect alterations with low VAF% limits the clinical applicability of such broader approaches being expensive and not always feasible [67].

Generally speaking, the SSNVs-based approach for ctDNA estimation may be affected by structural alterations occurring in the same genomic region. Additionally, it appears to be less than ideal in early-stage cancer settings due to the following: (1) the expected low somatic mutational rate; (2) the generally low shedding rate; and, consequently, (3) the low proportion of cfDNA fragments harboring somatic tumor-specific alteration, which requires continuous methodological optimization [31,68].

### 3.2. Genomics Analysis of the Circulating Tumor Fraction: Somatic Copy Number Alterations

Another NGS-based approach for calculating the cTF consists of the evaluation of the SCNAs (somatic copy number alterations and large-scale aneuploidies) in cfDNA using WGS. SCNAs are defined as chromosome-level changes, such as deletions or amplifications of large genomic regions, including both sub-chromosomal parts (i.e., focal CNAs) and entire chromosome arms (aneuploidy) [69]. Compared to SSNVs, the analysis of SCNAs may be more broadly applicable, as it is not dependent on a specific sequence variant and most cancers harbor arm-level CNAs [70].

Some WGS approaches for cTF calculation using SCNAs require a high-coverage depth of up to 100× [71,72]. While this deep-coverage WGS data analysis, which also provides allelic information for sequence variants, is considered the most established SCNA-based method for cTF estimation, it is cost-intensive and requires high-quality cfDNA, making its application in routine clinical pipelines challenging. To overcome these limitations, approaches based on low-pass and ultra-low-pass WGS (LP/ULP-WGS) or shallow WGS (sWGS), with a typical median depth of 0.1×–1×, were adopted as a rapid and low-cost method [73,74]. A number of bioinformatics teams have developed algorithms and computational approaches for SCNA analysis from LBx sWGS data, including ABSOLUTE [71], TITAN [72], QDNAseq [75], WisecondorX [76], SAMURAI [77], PRINCe [78], and ACE [79], among others. These tools are often integrated into custom pipelines to infer cTF. Adalsteinsson and colleagues developed ichorCNA, a ULP-WGS (0.1x) software package that predicts SCNA segments and estimates the tumor fraction (https://github.com/broadinstitute/ichorCNA (accessed on 10 November 2025)). With a lower limit of detection of 0.03, ichorCNA achieves a sensitivity of 0.95 for detecting the presence of a tumor and a specificity of 0.91 for classifying a healthy donor. At a tumor fraction threshold of 0.1, the approach reaches 91% sensitivity and 100% specificity [80]. The successful application of sWGS for detecting aneuploidy in NIPT, which identifies fetus-derived DNA in maternal plasma, paved the way for similar methodological approaches for detecting tumor-associated CNAs [81]. Interestingly, several authors have reported the incidental detection of presymptomatic maternal occult malignancies in this setting [82]. In line with this, commercial kits, such as the Ion ReproSeq PGS™ preimplantation genetic testing kit (ThermoFisher, Waltham, MA, USA), coupled with ichorCNA software (https://github.com/broadinstitute/ichorCNA (accessed on 10 November 2025)), have been successfully used for ctDNA rate calculation [83]. Recently, Lakatos and colleagues developed the LiquidCNA tool for cTF estimation from sWGS, which also allows for the longitudinal tracking of SCNAs across samples from the same patient to characterize subclonal tumor evolution [84]. Other sWGS approaches for analyzing tumor-associated copy number changes include Plasma-Seq [73] and the ThruPLEX^®^ Tag-Seq Kit (Takara, Shiga, Japan) [85]. Interestingly, even third-generation sequencing approaches like Nanopore technology have been successfully used for cTF estimation. While long-read sequencing is generally not ideal for analyzing short cfDNA fragments, additional methodological steps (e.g., enrichment using a specific bead/sample ratio) have improved the detection of CNA profiles from plasma cfDNA [86]. ULP-WGS has been optimized for detecting cancer and estimating cTF, even when the expected tumor fraction is low (i.e., <20%). It is noteworthy, however, that researchers have reported a potential underestimation of the ctDNA rate in cases of theoretically high tumor fractions (i.e., metastatic settings). It has been proposed that this is due to the impact of copy-neutral loss of heterozygosity events that are indistinguishable from truly copy-neutral segments using the resolution of ULP-WGS, resulting in lower tumor fraction estimation [87,88]. Several ULP-WGS applications for ctDNA estimation are available for metastatic prostate [12] or breast cancers [11,80,83]. The ichorCNA algorithm has also been applied to hepatocellular carcinoma (HCC) with aggressive clinical behavior, although it appears not to be sensitive enough for early HCC detection [89,90].

It is important to note that cTF measured using SCNAs is based on quantitative alteration occurring on a genome-wide scale. When compared to the SSNVs-based method, the cTF rate may differ from the observed VAF%. This can be explained by the fact that VAF% is affected by small copy number variation events, especially if they do not occur in copy-number-neutral regions, making the two methods not always comparable [91]. Additionally, it has been reported that SCNAs may fail to provide a robust estimation of cTF due to the lack of wide aneuploidy and chromosomal instability in some tumor types [92,93].

A summary of genomics-based approaches adopted for cTF estimation is collected in Table 1.

### 3.3. Epigenomics Analysis: Methylation Pattern

DNA methylation is an epigenetic mark that plays a key role in gene expression regulation and in genomic stability. It typically involves the formation of 5-methylcytosine (5mC) through the covalent addition of a methyl group to a cytosine base, primarily in cytosine-guanine dinucleotide (CpG)-rich sequences [99]. More recently, analysis based on the 5-hydroxymethylcytosine (5hmC) has also gained attention. The 5hmC is an intermediate in the process of DNA demethylation consisting in a modified cytosine generated through the oxidation of 5mC [100]. DNA methylation occurs early in tumor cells, and its profile is dynamically regulated during tissue development and differentiation, resulting in tissue- and cell-specific gene expression [101]. The role of the methylation process in cancer development and progression is well documented, though many aspects remain unclear. Abnormal hypo- and hyper-methylation lead to novel oncogenic properties, primarily linked to tumor suppressor gene silencing and chromosomal instability [102]. These aberrations can be seen as follows: (1) a global hypomethylation profile, explained by the higher transcriptional activity of cancer cells; and (2) focal hypermethylation spots occurring at specific gene promoters [103]. The DNA methylation patterns that characterize tumor cells are also preserved in shedding ctDNA. The methylation status of the cfDNA has the theoretical advantages of being stable over solid tumor development and indicative of both cancer presence and tissue of origin. This is important in clinical practice, as some DNA methylation alterations are consistent across various cancer types, while others are unique to a specific cancer [104]. In this context, the term tumor methylated fraction (TMeF) can be used to refer to the cTF with cancer-specific methylated aberrations. The rationale for ctDNA quantification using methylation patterns is based on the existence of differentially methylated regions (DMRs) associated with tumors, used to differentiate cfDNA from cancer-derived ctDNA.

Overall, the value of detecting and quantifying TMeF has been described in the contexts of early cancer diagnosis (MCED), the monitoring of disease response, and progression [68,105,106,107]. Generally, the evaluation of epigenetic markers has been described as superior to SSNVs for early-stage cancer diagnosis [42,108,109]. For instance, the Galleri bisulfite methylation test, applied to 4077 patients, reported an overall sensitivity of 90.1% for the detection of cancer Stage IV but only 27.5% for Stage I and II [110], data that were confirmed in the SYMPLIFY study [111,112].

From a methodological perspective, similar to SSNVs, LBx studies adopting methylation analysis can employ either a targeted or a broader, whole-genome approach [113]. Additionally, methods fall into two main categories, conversion-based and conversion-free. Conversion of unmethylated cytosines to uracil using bisulfite treatment or enzymatic methods before PCR and sequencing is the most common technique for cfDNA methylation analysis. However, it requires chemical treatments that can lead to significant molecule loss and low sequencing efficiency [114]. Alternatively, non-conversion-based methods allow for the targeted capture of specific methylated DNA regions. Examples include the cell-free methylated DNA immunoprecipitation (cfMeDIP) and the cell-free methyl-binding domain (cfMBD). These approaches use 5mC antibodies to enrich methylated cfDNA fragments and identify methylation levels and TMeF through fluorescence [115,116]. Some authors have noted that capture-based, conversion-free methods are less sensitive in the analysis of hypomethylated regions, which may be relevant for determining the tissue of origin of ctDNA [28]. Most methylation-based quantification methods in LBx focused on identifying preselected panels of CpG sites, with differentiated methylation status known to be relevant in a specific clinical context. These approaches generally require prior tissue methylation profiling (tumor vs. normal) during the assay design stage [117]. Methylation-specific qPCR has been developed to analyze one or a few tumor-specific loci that show a correlation with clinical outcomes [118,119,120,121,122,123]. Successful applications have been described in HCC, where approaches effectively detected the presence of HCC and distinguished it from benign hepatic lesions [124]. The SEPT9 bisulfite- and PCR-based assay is FDA approved for colorectal cancer (CRC) screening [125]. However, meta-analyses suggest that the SEPT9 methylation test is inferior to existing fecal immunohistochemical assays in asymptomatic cohorts [126]. An example of a targeted digital PCR approach is the TriMeth assay, which targets three CRC-specific methylation markers (C9orf50, CLIP4, and KCNQ5) [127].

In addition to these successful examples, PCR-based or capture-based approaches can be unfeasible for large multiplex analysis of many targets and are not ideal for broad ctDNA quantitation [128]. Recently, an NGS-based assay covering more than 500 DMRs was developed and successfully applied in several studies [124,129]. This assay, named Northstar Response, compares the count of methylated cfDNA molecules at specific genomic locations, known to be hypermethylated in cancer tissue, to those in normal tissue. To accurately count the number of methylated molecules at each location, the assay leverages quantitative counting templates (QCTs) designed to amplify similarly to sample molecules during the PCR step [130]. Methylation-based quantitation is included in approved commercial kits (Galleri^®^, GRAIL, Epi proColon 2.0, Menlo Park, CA, USA). The Guardant Reveal^®^ assay (Guardant Health, Palo Alto, CA, USA) estimates cTF by evaluating 300 DMR sites using a bisulfite-free NGS approach. The cTF is estimated by normalizing the count of methylated molecules from cancer-specific DMRs with the count of methylated molecules from a matched control region using a proprietary algorithm.

Genome-wide shotgun massively parallel bisulfite sequencing is successfully used in several tumor types with the theoretical advantage of requiring only low-depth sequencing [131,132]. This is because the methylation changes are pervasive in the genomes and can be evaluated with a lower number of reads compared to other structural or sequence changes. Some authors have demonstrated the feasibility of this approach with a sequencing depth as low as 10 million reads, making it a relatively cost-effective option [131]. NGS applied to methylation analysis requires specific bioinformatics pipelines, such as Methy-Pipe [133], which counts methylated cytosines along the sequencing reads. Methylation-sensitive restriction enzyme digestion followed by sequencing (MRE-Seq) has also proven to be useful for investigating the cancer signal origin (CSO) using a deep neural network (DNN) analysis in CRC and lung cancer [134]. Overall, analytical workflow that allows for the simultaneous analysis of sequence variants (SSNVs) and methylation status of ctDNA are ideal and the most attractive [135]. Examples of long-read sequencing (third-generation sequencing) are also available in the literature. For instance, the Oxford Nanopore technology directly analyses the native cfDNA molecule without the need for PCR or enrichment steps and provides information about both methylation status and genomic alterations simultaneously.

### 3.4. Epigenomics Analysis: Fragmentomics

The term “cfDNA fragmentome” refers to the genome-wide profile of cfDNA fragments, including size, distribution, breakpoint locations, and end motif profile. This information provides a comprehensive view of the genomic, epigenomic, and transcriptomic state of the cfDNA, being based on nucleosome positioning with respect to nucleosome center, chromatin structure, and nuclease activity during cell death [136]. The fragmentation of cfDNA is a highly conserved process across mammals, influenced by the nucleosome structure that protects the DNA from cleavage. It is known that cfDNA in human plasma is nonrandomly fragmented by caspase enzymes [137]. In fact, the association of DNA molecules with nucleosomes, the basic unit of DNA packaging, avoids the fragmentation process and protects the nucleic acid. The activity of caspase explains the common profile observed in cfDNA. In healthy subjects, cfDNA fragments generally showed a mode of ~167 bp, in which ~147 bp are wrapped and protected around the nucleosome, with a 10 bp periodicity corresponding to a turn of the DNA helix, and 20 bp consisting of a linker region site of cleavage [22,138,139,140,141]. The cfDNA profile of patients with cancer is characterized by a significant increase in shorter fragments (size range of 20 to 150 bp) and a length reduction predominantly at the C-end [131,142,143]. This can be explained by a more open chromatin state in cancer cells due to greater transcriptional activity, which makes more sites accessible for cleavage [144]. Variability in fragment size distribution has been observed across different cancer types. Mouliere and colleagues observed an enrichment of shorter fragments (20–150 bp) in plasma samples obtained from patients affected by melanoma, breast, ovarian, lung, CRC, and cholangiocarcinoma cancers (called “high ctDNA cancers”), but not in renal, glioblastoma, bladder, or pancreatic ones (called “low ctDNA cancers”) [145]. Additionally, a link between fragmentomic patterns and tissue-specific gene expression has been demonstrated [145,146,147]. The abovementioned two main characteristics of ctDNA, the shortening of fragments and the lowered C-end predominance, have been adopted to distinguish and quantify cTF [137]. While methods like capillary electrophoresis and ddPCR can provide general information on fragment length, a broader approach like WGS allows for a more sensitive assessment of the global cfDNA fragmentation pattern [148,149]. Recently, targeted approaches have also been developed, focusing on fragmentation patterns within the coding regions of oncogenes and oncosuppressors [150]. Methods to evaluate fragmentomics and infer cTF include the following: (1) deep sequencing with molecular barcodes and bioinformatics-based error correction; (2) size selection strategies for the enrichment of a subpopulation of fragments; and (3) low-coverage WGS combined with machine learning analysis. Most studies in the literature have focused on the selection of short fragments (90–150 bp) to enrich for the ctDNA fraction, based on differences in fragment size distribution across a genome [144,151]. The size selection approach has also been used for genomic or methylation-based studies, offering the integration of multiple omics in the same experiment, with the aim of improving the sensitivity of a downstream assay. Wang and colleagues, for example, adopted an optimized cfMeDIP-seq approach to gather information on both methylation and fragmentation patterns in breast cancer [152]. Mouliere and co-authors developed a “double step” of cfDNA size selection. At first, they select 90–150 bp fragments of cfDNA from an agarose cassette, which were then used as an input for sWGS with CNV analysis, followed by a second size selection based on in silico NGS read filtering [153]. Similar enrichment methods have been achieved by excising bands of an appropriate size from polyacrylamide gels, resulting in a significant enrichment of the ctDNA fraction, from 2.5-fold to 9.1-fold [154]. Enrichment methods can also be applied to sequencing reads using bioinformatics pipelines, such as with the INVAR, that integrate variant reads and signal enrichment based on biological fragmentation patterns of ctDNA [155]. Other enrichment techniques include the single-strand binding polymerase chain reaction (SSB-PCR), probe detection systems based on IV endonuclease, rolling circle amplification, and hybrid capture [156]. Recently, cfDNA fragmentome analysis obtained using an LP-WGS combined with the machine learning pipeline DNA evaluation of fragments for early interception (DELFI) demonstrated high sensitivity for ctDNA quantification and early detection in several clinical settings, such as ovarian [136], lung [157,158], and liver [159], among others [69,152,160,161,162]. To note, Markus and colleagues reported the utility of the fragmentomic analysis in ovarian cancer samples not only in the evaluation of fragments shorter than mono-nucleosomes (~167 bp), but also shorter than di-nucleosomes (~240–330 bp). They also observed an enrichment of DNA fragments that start and end at the border or within the nucleosome core in the ctDNA rate. This information sheds light on the complexity of the biological characteristics of ctDNA fragmentation, underlining the utility of an integrated approach to detect tumor-derived fragments [94]. Further information related to ctDNA fragments, such as the analysis of ctDNA-preferred ends, has been explored in multiple studies and relies on the identification of tumor-associated cfDNA-preferred end coordinates and abundance using the NGS approach [163,164]. The integration of both fragmentomic information and epigenetic cfDNA features seems to be a good marker of early cancer detection and residual disease monitoring, boosting the diagnostic capabilities [165]. Furthermore, by combining fragmentomic information and SCNAs, Janke and colleagues demonstrated the utility of multi-omics data integration in the prediction of therapy response in advanced lung cancer at different time points [166].

A summary of epigenomics-based approaches adopted to cTF estimation is collected in Table 2.

## 4. Integration of Multi-Omics Data

The successful implementation of a multimodal strategy in LB relies on several sophisticated computational tools capable of integrating two or more different layers of biological information. These methods advance beyond the single-marker analysis using machine learning (ML) and artificial intelligence (AI) approaches to decode the complex biological signatures that distinguish ctDNA from cfDNA. Given the complexity of cancer biology and maximizing the probability of cancer detection, cTF estimations from each biological layer can be combined into a single and more robust score, potentially weighted by the reliability or strength of the signal derived from each modality for a given cancer type. In this context, examples of ML and AI tools include random forests [167], support vector machines [168], and deep neural networks [169]. These classifiers can be trained on several input biological features extracted from genomics (SSNVs, SCNAs), epigenomics (methylation patterns), and fragmentomics (size distribution, end motifs) datasets to improve the sensitivity and specificity of cancer detection and cTF quantification. Such data integration models have proven to be particularly effective for challenging LB scenarios like ctDNA detection in the early-stage setting [170,171]. Overall, two main ML and AI strategies can be identified. An approach consists of building scores or classifiers based on the combination of individual omic layer score outputs to make an overall prediction. Moldovan and co-authors developed an ML classifier pipeline that combines cfDNA genomic (specifically, SCNAs using ichorCNA) and fragmentomic (specifically, using the open pipeline called FrEIA) features to reach a sensitive detection of tumor-derived cfDNA starting from the same sWGS dataset [172]. The authors evaluated four supervised ML approaches (k-neighbors, logistic regression, random forest, and support vector classifier) starting from four genomics and epigenomics cfDNA datasets, identifying that logistic regression provided the highest estimation of classification performance. A multi-class prediction model trained by a support vector classifier was adopted by Siejka-Zielinska and co-authors using methylation data and fragmentomics analyses and was successfully applied in the early HCC and pancreatic ductal adenocarcinoma settings [173]. Another approach for integrating omic-specific features is to use ensemble classifiers [174,175]. For instance, the ensemble THEMIS classifier was applied to the integration of four input features (methylated fragment ratio, fragment size index, chromosomal aneuploidy, and fragment end motif) to improve the ctDNA detection specificity and sensitivity over the individual features alone [176]. An additional example is the ML method named lung cancer likelihood in plasma (Lung-CLiP) that includes targeted sequencing of plasma cfDNA, matches leukocyte DNA, and integrates SSNV calling (leverages specific biological and technical features as cfDNA fragment size, the gene affected, and the likelihood of CHIP) with the genome-wide SCNAs analysis [177]. In addition to AI and ML bioinformatics pipelines, it is relevant to note that cutting-edge long-read sequencing technologies provide the opportunity to obtain multi-modal data from a single sequencing experiment, analyzing fragmentomics, methylomics, as well as genomic alterations [178,179]. Despite the current limitations of such platforms, primarily related to base-calling accuracy and bioinformatics pipeline implementation, it stands to reason that the continued evolution of technology and algorithms will unlock the full potential of nanopore and PacBio technology for cfDNA-based liquid biopsy analyses.

**Figure 1:**
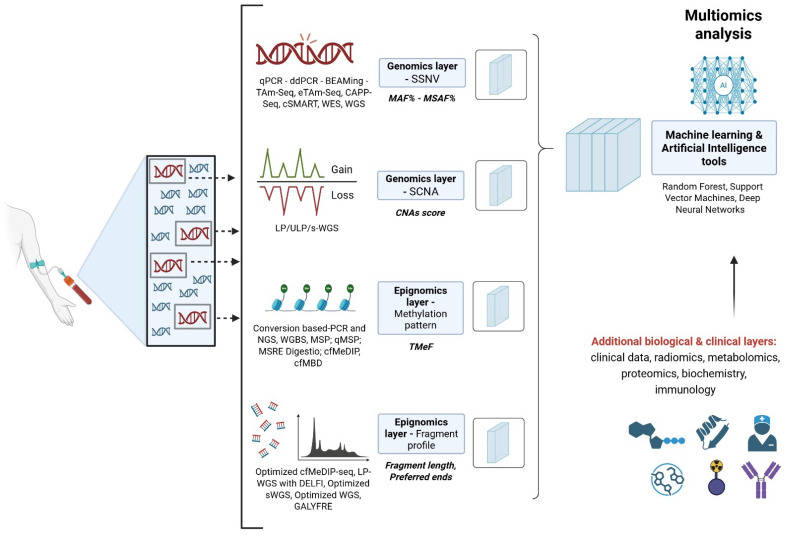
Integration of multi-omics layers. The Figure shows the schematic multi-modal integration of the genomics and epigenomics strategies for ctDNA estimation. For both genomics and epigenomics layers, we reported the main methodological approaches available (e.g., ddPCR, WGS), the investigated molecular features (e.g., SSNV, SCNA), and the output variable type (e.g., MAF%, TMeF). Examples of machine learning approaches for data integration were also reported. Created in https://BioRender.com.

Looking to the ultimate goal of cancer research in terms of prevention, early detection, personalized paths of treatments, and optimization of disease monitoring, AI approaches can also be adopted for the integration of multiomics LB data for additional clinical, pathological (e.g., biochemistry), and radiological information (Figure 1) [180]. The direction of ML adoption consists of the shift from predictive to generative models that can elaborate on new entities, such as digital human avatars, that serve as integrative predictive models for cancer patients to support clinicians in improving decision-making processes [181,182].

## 5. Translational Outlook and Standardization Challenges

The great research and technical improvements of LB approaches hold immense promise for their robust application in clinical oncological settings. However, several hurdles must be overcome to fully move these powerful technologies from the research bench to routine clinical care. Indeed, their translational value is significantly hampered by the lack of standardized protocols and regulatory oversight. Moreover, current approaches face limitations in terms of clinical applicability, and also mainly due to cost, data handling requirements, and turnaround time [183,184]. In both research and clinical contexts, a considerable inter-laboratory variability has clearly emerged in cfDNA processing, sequencing, and proprietary bioinformatic pipelines used for ctDNA estimation and, generally speaking, for molecular data interpretation. Some of the LB technologies and assays described here benefit from regulatory approval status from the FDA and European CE-IVD. These regulatory frameworks differentiate clinically actionable and reimbursed tests from emerging research tools. To date, regulatory approvals mainly cover SSNV-based (both PCR and NGS) and methylation-based assays (e.g., cobas^®^ *EGFR* Mutation Test v2, FoundationOne^®^ Liquid CDx, Epi pro-Colon^®^) [17,31,35,48,53,65,97,98,110,125,131]. Other promising assays lack the same rigorous regulatory standing, even if they can be offered as a laboratory-developed test (LDT) in clinical laboratory improvement amendments (CLIA) or the European Union’s in vitro diagnostic medical device regulation (IVDR)-certified labs (Galleri^®^, Guardant Reveal^®^). Techniques such as the SCNA-based method and fragmentomics are currently suitable for the research setting. Their current lack of regulatory standardization results in a significant inter-laboratory variability, insufficient clinical validation from large prospective trials, and, consequently, no effective path to a widespread clinical application.

## 6. Concluding Remarks

The appropriate estimation of cTF is critical for the reliability of the analytical test and for the clinical utility of LBx data. The variability in the methodological approach adopted to estimate the ctDNA rate highlights the capacity to evaluate different features of the ctDNA. The choice of the best methodology depends on the specific cancer biology. The various “omics” approaches (genomics, epigenomics, and fragmentomics) offer a unique way to infer cTF, with their own strengths and limitations. Genomics focuses on the specific SSNVs that are most effective when a target, patient-specific mutation is known from a TBx, but they may be less useful in tumors with low mutation rates. Conversely, SCNAs are broadly applicable, since most cancers have widespread chromosomal aberrations. However, both methods can be challenged by CHIP or insufficient ctDNA shedding. Because methylation patterns can appear early in tumor development, methylation-based methods are particularly promising for early cancer detection. The signal can be stronger and more widespread than a single SSNV, making it a powerful tool for quantification, but it requires specialized assays and analysis pipelines [49]. Fragmentomics can provide insights into tumor biology and is increasingly being combined with machine learning to improve cTF estimation sensitivity. Under the selective pressure of therapy, the biological behavior of tumor cells can lead to evolving genomic and epigenomic profiles over serial sampling. Consequently, the molecular features (e.g., SSNV, CNAs) adopted for cTF calculation in a specific clinical time point could not be equal in another one. Given the complexity and variability of tumor biology, the integration of multiple omics data, often derived from a single sequencing run, is the most promising strategy. The goal is to move towards a multi-modal liquid biopsy that provides a reliable, “all-in-one”, solution for cancer management.

## Figures and Tables

**Table 1:** Summary of genomics approaches applied to circulating tumor fraction estimation.

ctDNA Biological Feature	Technology	Method	Assay	Molecular Target	Technical Specification	Cost Tier and Regulatory Standard	Advantages	Disadvantages	Refs.
SSNV	PCR-based (MAF%)	qPCR	ARMS; superARMS PCR; PNA-LNA PCR clamp; AS-NEPB-PCR; COLD PCR	Hotspot (SNVs, indels)	LoD: 0.01–1% Specificity: >99% cfDNA input: 1–50 ng	Low; LDT, CLIA/IVDR	Fast and robust for targeted monitoring; cost-effective; ease of use; useful as prognostic/predictive biomarker.	Limited multiplexing from one to few targets; requires TBx for personalized approach; lack of tumor heterogeneity analysis over time; time-consuming for custom design; may not be ideal for early-stage cancers with low mutation rates.	[24,94,95,96]
		dPCR	ddPCR; BEAMing	Hotspot (SNVs, indels)	LoD: 0.01–0.1% Specificity: >99%; cfDNA input: 1–25 ng	Low–Medium; LDT, CLIA/ IVDR, FDA-approved (e.g., cobas^®^ *EGFR* Mutation Test v2)	Fast and robust for targeted monitoring; cost-effective; high sensitivity; absolute quantification; useful as prognostic/predictive biomarker.		[17,24,31]
SSNV	NGS-based (MSAF%)	Targeted	TAm-Seq, eTAm-Seq, CAPP-Seq, cSMART	Hotspot and whole gene (SNVs, indels)	LoD: 0.02–0.5% Specifici-ty: >98%; cfDNA input: 10–50 ng cfDNA	Medium–High; RUO, LDT, CLIA/IVDR, FDA-approved (e.g., FoundationOne^®^ Liquid CDx)	TBx-free; high multiplexing capacity; prediction of molecular signature; high sensitivity; robust for targeted monitoring; useful as prognostic/predictive biomarker.	Expensive due to deep coverage needed; long TAT; analytical variability related to gene panel choice; may not be ideal for early-stage cancers with low mutation rates.	[35,49,55,97,98]
		Untargeted	WES; WGS	Coding regions, intron-exon junction; Whole genome (SNV, indels)	LoD: 5–10% Specificity: N/A cfDNA input: >50 ng cfDNA	High; RUO	TBx-free; mutation discovery; prediction of molecular signature; broader applicability.	Lower sensitivity; need for bioinformatics skills and optimized pipeline; not ideal for tumors with low somatic mutational rate and lower shedding; need for validation, research only; cost-intensive and requires high-quality cfDNA.	[67,74]
SCNAs	NGS	Untargeted	LP/ULP/s-WGS	Whole genome (CNA)	LOD: ~3%; Specificity: ~90%. cfDNA input: 5–20 ng	Medium; RUO, LDT	TBx-free; SCNA discovery; cost-effective; broader applicability.	Need for bioinformatics skills and optimized pipeline; need for validation; not ideal for tumor lacking. chromosomal instability.	[12,80,83]

Footnotes: SSNVs: single sequence nucleotide variants; SCNAs: somatic copy number alterations; PCR: polymerase chain reaction; qPCR: quantitative-PCR; ARMS/superARMS PCR: amplification refractory mutation system PCR; PNA-LNA PCR clamp: peptide nuclei acid-locked nucleic acid PCR clamp; AS-NEPB-PCR: allele-specific non-extendable primer blocker PCR; COLD PCR: co-amplification at lower denaturation temperature PCR; SNV: single-nucleotide variant; LOD: limit of detection; LDT: laboratory-developed test; CLIA: clinical laboratory improvement amendments; IVDR: in vitro diagnostic medical device regulation; RUO: research use only; TAT: turnaround time; TBx: tumor biopsy; ddPCR: droplet digital PCR; BEAMing: beads, emulsions, amplification, and magnetics; CNA: copy number alteration; TAm-Seq: tagged-amplicon deep sequencing; eTAm-Seq: enhanced TAm-Seq; CAPP-Seq: cancer personalized profiling by deep sequencing; cSMART: single-molecule amplification and resequencing technology; WES: whole-exome sequencing; WGS: whole-genome sequencing; LP: low pass; ULP: ultra-low pass; and sWGS: shallow WGS.

**Table 2:** Summary of epigenomics approaches applied to circulating tumor fraction estimation.

ctDNA Biological feature	Technology	Method	Assay	Molecular Target	Technical Specification	Cost Tier and Regulatory Standard	Advantages	Disadvantages	Refs.
TMeF	Conversion-based	Bisulfite	PCR, NGS	From one to a few targets	LoD: ~0.1–0.5% Specificity: ~91.5%; cfDNA input: 10–20 ng	Low–Medium; LDT, CLIA/IVDR, FDA-approved (Epi pro-Colon^®^)	High sensitivity; can be combined with several downstream methods	Requires TBx for a personalized approach; chemical treatments that can lead to molecule loss and bias; need for bioinformatics skills and optimized pipeline.	[67,125]
			WGBS	Whole methylome	LoD: varies by clinical stage; Specificity: >99.5%; cfDNA input: 10–50 ng	High; LDT, CLIA/IVDR (e.g., Galleri^®^, Guardant Reveal^®^)	Broader applicability; need for low-depth sequencing; relatively cost-effective; biomarker discovery; detect early cancer and tissue of origin	Requires technical skills; need for validation, research only; high sequencing and computational costs; need for high-quality DNA; need for bioinformatics skills and optimized pipeline.	[110,131]
	not conversion-based	Enzymatic, antibody	MSP; qMSP; MSRE Digestio; cfMeDIP, cfMBD	From one to a few targets From one to a few targets	LoD: ~1% Specificity: ~92%; cfDNA input: ~10–30 ng	Medium; RUO	Relatively easy to use and cost-effective, detect early cancer and tissue of origin	Can be less sensitive in hypomethylated regions detection; requires TBx for personalized approach; PCR bias and optimization challenges; Antibody specificity bias; limited by enzymatic recognition sites available	[115,116]
Fragment length analysis	In silico enrichment-based	WGS	Optimized cfMeDIP-seq, LP-WGS with DELFI	Whole cfDNA	LoD: varies according to enrichment factor; Specificity: >98%; cfDNA input: 5–20 ng	Medium; RUO, LDT	Broader applicability; highly sensitive; allows multi-omics integration	Need for bioinformatics skills and optimized pipeline; need for validation, research use; need for optimized protocols for enrichment	[144,154,157]
	Hybrid enrichment approaches	NGS and CE	Optimized sWGS						
Preferred ends	NGS	WGS	Optimized WGS, GALYFRE	Whole cfDNA	LoD: Not applicable (re-search stage); Specificity: >70; cfDNA input: ~10 ng		Broader applicability; requires a limited depth of sequencing and a low amount of input DNA	Need for bioinformatics skills and optimized pipeline; need for validation, research use; need for tumor vs. normal setting.	[22]

Footnotes: PCR: polymerase chain reaction; LoD: limit of detection; LDT: laboratory-developed test; CLIA: clinical laboratory improvement amendments; IVDR: in vitro diagnostic medical device regulation; RUO: research use only; TBx: tumor biopsy; WGS: whole-genome sequencing; MSP: methylation-specific PCR; qMSP quantitative methylation-specific PCR; MSRE: methylation-sensitive restriction enzyme digestion; MeDIP: methylated DNA immunoprecipitation; WGBS: whole-genome bisulfite sequencing; CE: capillary electrophoresis; and GALYFRE: genome-wide analysis of fragment ends.

## Data Availability

No new data were created or analyzed in this study. Data sharing is not applicable to this article.

## Abbreviations

The following abbreviations are used in this manuscript:

ctDNA	Circulating tumor DNA
cfDNA	Cell-free DNA
cTF	Circulating tumor allele fraction
LBx	Liquid biopsy
CTCs	Circulating tumor cells
cfRNA	Cell-free RNA
NIPT	Non-invasive prenatal testing
MRD	Minimal residual disease
MCED	Multicancer early detection
TBx	Tissue solid biopsies
FDA	Food and Drug Administration
eccDNA	Circular DNA
SNVs	Single-nucleotide variants
CNAs	Copy number alterations
NGS	Next-generation sequencing
UMIs	Unique molecular identifiers
SSNVs	Somatic single-nucleotide variants
VAF	Variant allele frequency
MAF	Mutant allele frequency
ddPCR	Droplet digital PCR
BEAMing PCR	Beads, emulsions, amplification, and magnetics PCR
AS-NEPB-PCR	Allele-specific non-extendable primer blocker PCR
PNA-LNA PCR clamp	Peptide nucleic acid-locked nucleic acid PCR clamp
COLD PCR	Co-amplification at lower denaturation temperature PCR
PCR	Polymerase chain reaction
LOD	Limit of detection
LDT	Laboratory-developed test
CLIA	Clinical laboratory improvement amendments
IVDR	European Union’s in vitro diagnostic medical device regulation
RUO	Research use only
CT	Computed tomography
CHIP	Clonal hematopoiesis of indeterminate potential
TAm-Seq	Tagged-amplicon deep sequencing technology
eTAm-Seq	Enhanced TAm-Seq
CAPP-Seq	Cancer personalized profiling by deep sequencing
cSMART	Single-molecule amplification and resequencing technology
MSAF	Maximum somatic allele frequency
GEMINI	Genome-wide mutational incidence for non-invasive detection of cancer
WES	Whole-exome sequencing
WGS	Whole-genome sequencing
SCNAs	Somatic copy number alterations
LP/ULP-WGS	Low-pass/ultra-low-pass WGS
sWGS	Shallow WGS
HCC	Hepatocellular carcinoma
qPCR	Quantitative-PCR
ARMS/superARMS PCR	Amplification refractory mutation system PCR
TAT	Turnaround time
5mC	5-methylcytosine
CpG	Cytosine-guanine dinucleotide
5hmC	5-hydroxymethylcytosine
TMeF	Tumor methylated fraction
DMRs	Differentially methylated regions
cfMeDIP	Cell-free methylated DNA immunoprecipitation
cfMBD	Cell-free methyl-binding domain
CRC	Colorectal cancer
QCTs	Quantitative counting templates
MRE-Seq	Methylation-sensitive restriction enzyme digestion followed by sequencing
DNN	Deep neural network
SSB-PCR	Single-strand binding polymerase chain reaction
DELFI	DNA evaluation of fragments for early interception
MSP	Methylation-specific PCR
qMSP	Quantitative methylation-specific PCR
MSRE	Methylation-sensitive restriction enzymes digestion
WGBS	Whole-genome bisulfite sequencing
CE	Capillary electrophoresis
GALYFRE	Genome-wide analysis of fragment ends
ML	Machine learning
AI	Artificial intelligence
